# The enhanced recovery after surgery (ERAS) program in liver surgery: a meta-analysis of randomized controlled trials

**DOI:** 10.1186/s40064-016-1793-5

**Published:** 2016-02-29

**Authors:** Wei Song, Kai Wang, Run-jin Zhang, Qi-xin Dai, Shu-bing Zou

**Affiliations:** Department of Hepatobiliary Surgery, The Second Affiliated Hospital of Nanchang University, No.1 Minde Road, Nanchang, China

**Keywords:** Liver surgery, Enhanced recovery after surgery, Meta-analysis

## Abstract

The enhanced recovery after surgery (ERAS) program aims to attenuate the surgical stress response and decrease postoperative complications. It has increasingly replaced conventional approaches in surgical care. To evaluate the benefits and harms of the ERAS program compared to conventional care in patients undergoing liver surgery. We searched the MEDLINE, PubMed, EMBASE and Cochrane databases. All RCTs that compared the ERAS program with conventional care were selected. Four RCTs were eligible for analysis, which included 634 patients (309 ERAS vs. 325 conventional). Overall morbidity (RR 0.67; 95 % CI 0.48–0.92; p = 0.01), primary length of stay (WMD −2.71; 95 % CI −3.43 to −1.99; p < 0.00001), total length of stay (WMD −2.10; 95 % CI −3.96 to −0.24; p = 0.03), time of functional recovery (WMD −2.30; 95 % CI −3.77 to −0.83; p = 0.002), and time to first flatus (SMD, −0.52; 95 % CI −0.69 to −0.35; p < 0.00001) were significantly shortened in the ERAS group. Quality of life was also better in the ERAS group. However, no significant differences were noted in mortality, readmission rates, operative time and intraoperative blood loss. The ERAS Program for liver surgery significantly reduced overall morbidity rates, accelerated functional recovery, and shortened the primary and total hospital stay without compromising readmission rates. Therefore, ERAS appears to be safe and effective. However, the conclusions are limited because of the low methodological quality of the analyzed studies. Further studies are needed to provide more solid evidence.

## Background

Liver resection refers to the removal of a portion of the liver. Elective liver resection is performed mainly for benign and malignant liver tumors (Belghiti et al. 1993). The common reasons for liver resection are hepatocellular carcinoma and colorectal liver metastases. Liver resection has long been considered a major abdominal surgery and is associated with high morbidity and mortality. With the advancement of operative skills and equipment, overall mortality rates below 4 % can be achieved, but morbidity rates remain high at 10–40 % (Finch et al. 2007; Reissfelder et al. 2011).

Enhanced recovery after surgery (ERAS) or Fast track surgery (FTS), a new perioperative care, was developed to improve surgical outcomes and has gradually replaced conventional approaches in surgical care. The multi-modal therapy initiated by Kehlet et al. in 1995 (Bardram et al. 1995) incorporates a number of techniques that attenuate surgical stress response and accelerate recovery by preserving bodily composition and organ functions. According to the recommendations of the ERAS working group, ERAS programs consist of 17 different items, including preoperative, perioperative and postoperative measures. Techniques include preoperative counseling and feeding, epidural anesthesia, minimally invasive incisions, optimal pain control, fluid overload prevention, no routine use of drains, and aggressive postoperative rehabilitation (Lassen et al. 2009). Therefore, ERAS requires cooperation between doctors, anesthetists, nurses and physical therapists (Kehlet and Dahl 2003; Kehlet and Wilmore 2002).

ERAS programs have been adopted by a myriad of specialties, including colorectal surgery, gynecology, urology and orthopedic surgery (Nelson et al. 2014; Koo et al. 2013; Jones et al. 2014). ERAS programs have also been applied in liver surgery. However, there is no official statement or guidelines of ERAS Society regarding liver surgery, so the present centers only adopt inspired guidelines of ERAS program. ERAS programs for liver surgery seem to have positive effects (van Dam et al. 2012; Sánchez-Pérez et al. 2012; Dunne et al. 2014), but these effects have not been extensively studied. Two systematic reviews also showed a reduction of LOS in liver surgery (Coolsen et al. 2013; Hughes et al. 2014). However, such meta-analyses were performed with a limited number of poor quality RCTs. Recently, a meta-analysis (Lei et al. 2014) showed an overall reduction in morbidity and LOS for patients in ERAS programs after liver surgery. However, mortality and readmission rates were not analyzed. Furthermore, the studies did not take into account the number of ERAS items.

This paper evaluates the beneficial and harmful effects of ERAS programs compared to conventional care in patients undergoing liver surgery. This meta-analysis was executed in accordance with Cochrane guidelines (Higgins and Green 2011).

## Methods

### Search strategies

We searched MEDLINE, PubMed, EMBASE, and the Cochrane Central Register of Controlled Trials (CENTRAL). Search terms included perioperative care, preoperative care, postoperative care, convalescence, ERAS, fast track, enhanced recovery, and enhanced rehabilitation, combined with liver, hepatic, hepato-, resection, segmentectomy, and hepatectomy. To ensure a more complete meta-analysis, we used a maximally sensitive search for RCTs according to the Cochrane Highly Sensitive Search Strategy. Systematic reviews and meta-analyses were manually retrieved and served as references for included studies. Searches were completed until June 2015. No language restrictions were applied. There is no protocol in this meta-analysis.

### Inclusion criteria

The inclusion criteria were (1) RCTs comparing any type of ERAS program with conventional care in patients undergoing liver resection for malignant or benign disease and published in peer reviewed indexed journals; (2) patients undergoing elective, open or laparoscopic, resection of any portion of the liver; (3) studies that compared ERAS programs with conventional care and describe an ERAS program with at least seven items in ERAS groups and at most two items in the conventional groups; and (4) primary outcomes included mortality, overall morbidity (major and minor), overall hospital stay (primary and total), readmissions rates and functional recovery, while secondary outcomes included time to first flatus, operative time, intraoperative blood loss, and quality of life. Studies reported at least two of the primary outcomes.

### Exclusion criteria

The exclusion criteria were (1) non-randomized controlled trials; (2) studies that were non-elective or liver transplantation surgery; (3) studies in which it was impossible to obtain available data; and (4) duplicate publications between authors or centers.

### Data extraction and assessment of risk of bias

The data were independently extracted using a specifically designed data extraction form, which included study characteristics, patient characteristics, methodological quality and primary and secondary outcomes.

Study characteristics included study design, name of first author, sample size information, number of ERAS items, ASA classification, and follow-up time. Patient characteristics included number of patients, gender, male-to-female ratio, age at diagnosis and type of surgery. ERAS items were also extracted. ERAS programs should include 17 items according to ERAS group recommendations (Lassen et al. 2009). To better determine the efficacy of ERAS items and reduce clinical heterogeneity, we required that ERAS programs have at least seven items and that conventional procedures have at most two items to be sufficient for comparison, according to the Cochrane review (Spanjersberg et al. 2011). Primary outcomes included mortality, overall morbidity (major and minor), overall hospital stay (primary and total), readmissions rates, and functional recovery. Secondary outcomes included time to first flatus, operative time, intraoperative blood loss, and quality of life. Overall morbidity was divided into major and minor complications. Major complications included hepatic failure, biliary fistula, bleeding, intra-abdominal abscess, pleural effusion, ascites, prolonged ileus, need for reoperation, incisional hernia and adhesions. Minor complications included pulmonary infection, incisional infection, urinary tract infection, deep vein thrombosis and retention of urine. Mortality was separately evaluated. Overall hospital stay included primary hospital stay (PHS) and total length of stay (TLOS). TLOS included primary hospital stay and additional days for patients who were readmitted.

RCT quality was evaluated in accordance with the Cochrane Collaboration’s risk of bias tool (Higgins and Green 2011). This included an assessment of random sequence generation, allocation concealment, blinding of outcome assessors, blinding of participants and personnel, incomplete outcome data, selective reporting of outcomes and other biases. The bias of each item was graded as low, high or unclear.

### Statistical analysis

The meta-analysis was conducted using RevMan version 5.3 software (provided by Cochrane Collaboration). Heterogeneity was measured using Q tests and I^2^, and p < 0.10 was determined as significant (Higgins and Thompson 2002). If there was no or low heterogeneity (I^2^ < 25 %), then the fixed-effects model was used. Otherwise, the random-effects model was used. If significant heterogeneity existed, subgroup or sensitivity analyses were performed to decipher the reasons. The risk ratio (RR) was calculated for dichotomous data, and weighted mean differences (WMD) or standard mean differences (SMD) were used for continuous variables. Both differences were presented with 95 % CI. For continuous variables, if data were presented with medians and ranges, then we calculated the means and SDs according to Hozo et al. (2005). If the study presented the median and interquartile range, the median was treated as the mean, and the interquartile ranges were calculated using 1.35 SDs, as described in the Cochrane handbook.

## Results

### Search results

**Fig. 1:**
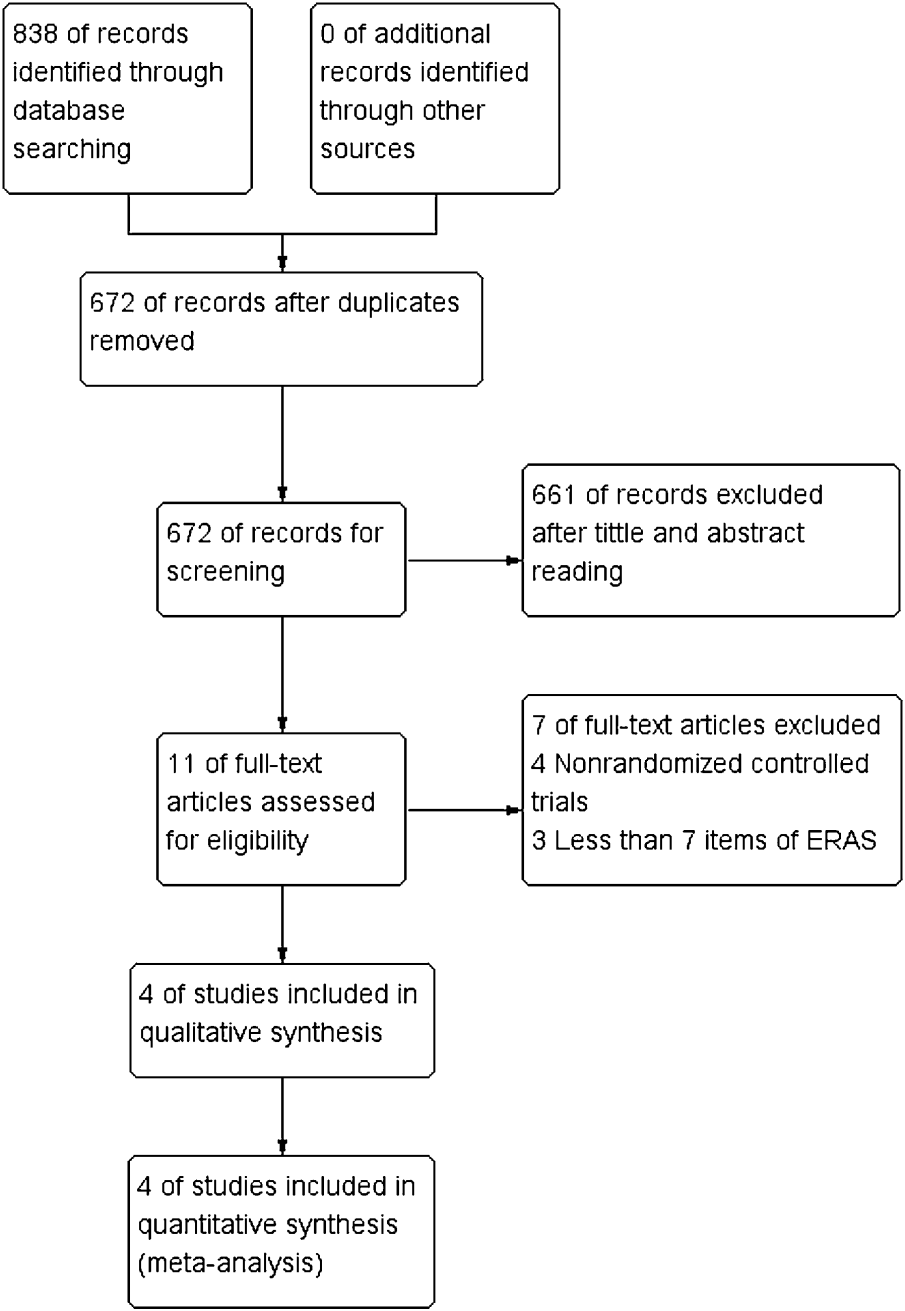
Search strategy

In total, 838 records were retrieved from the initial literature search. After the removal of duplicates (166 records), we identified 672 records by screening titles and abstracts. 661 articles were excluded, leaving 11 articles for further evaluation. Subsequently, seven articles were excluded by full-text reading, including three that had less than seven ERAS items, and four non-randomized controlled trials. Eventually, four RCTs were included in the meta-analysis (He et al. 2015; Jones et al. 2013; Lu et al. 2014; Ni et al. 2013). No additional records were identified (Fig. 1).

### Characteristics of the included studies

The four studies included 634 patients in total. Three hundred and nine patients received ERAS care, and 325 patients received conventional care. Detailed patient characteristics are shown in Table 1, and baseline study characteristics were similar.

**Table 1:** Characteristics of the included studies

Trail	Year	Number of patients		Age (years) mean ± SD/median (range)		Gender (M/F)		Follow-up (months)	ERAS items	ASA I/II (%)	
		ERAS	CC	ERAS	CC	ERAS	CC			ERAS	CC
He et al. (2015)	2015	48	38	56.3 ± 16.3	60.4 ± 20.7	22/26	18/20	5	12	75	94
Jones et al. (2013)	2013	46	45	64 (27–83)	67 (27–84)	31/15	23/22	1	15	93	88
Lu et al. (2014)	2014	135	162	54 ± 11.4	52.6 ± 11.3	111/24	133/29	1	12	100	100
Ni et al. (2013)	2013	80	80	48.4 ± 15.6	50.1 ± 21.8	66/14	59/21	ns	13	100	100

*ERAS* enhanced recovery after surgery, *TC* conventional care, *ns* not specified

### Number of ERAS items used

ERAS programs incorporated 17 interventions according to the recommendation (Lassen et al. 2009). All RCTs used a large number of ERAS items, ranging from 12 to 15; thus, they were considered high quality. The exact items used are provided in Table 2.

**Table 2:** ERAS items used in the included studies

Study	Preoperative						Perioperative				Postoperative						
	Preoperative counseling	Pre-operative feeding	Symbiotics	No bowel preparation	No pre-medication	Fluid restriction	Perioperative high O2 concentrations	Prevention of hypothermia	Epidural anesthesia	minimal invasive incisions	No routine use of NG tubes	No use of drains	Early post-operative mobilization	Early post-operative feeding	No systemic morphine use	Standard laxatives	Early removal of urine catheter
He et al. (2015)	√	√	√	√		√		√			√	√	√	√	√		√
Jones et al. (2013)	√	√	√	√	√	√	√	√	√		√	√	√	√	√		√
Lu et al. (2014)	√	√	√	√		√		√		√	√	√	√	√			√
Ni et al. (2013)	√	√		√	√	√			√		√	√	√	√	√	√	√

### Methodological quality of studies

**Fig. 2:**
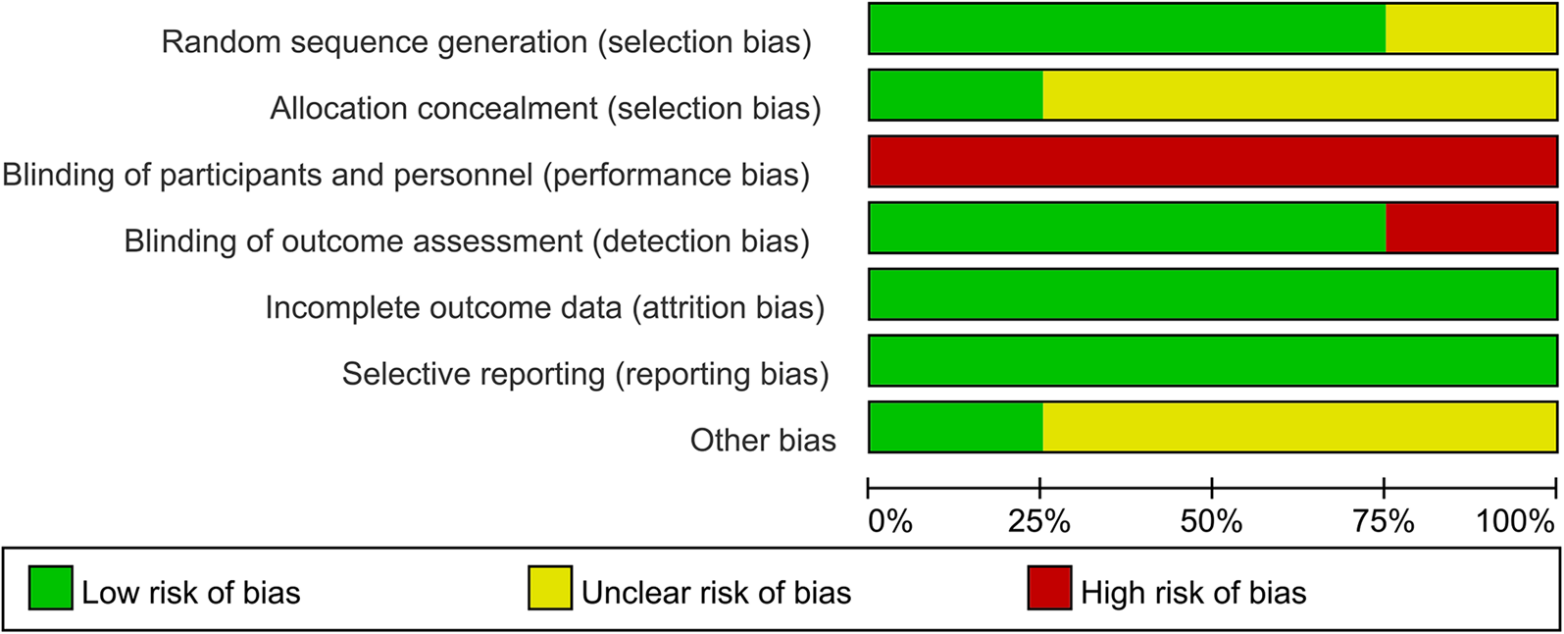
Risk of bias graph of all included studies

**Fig. 3:**
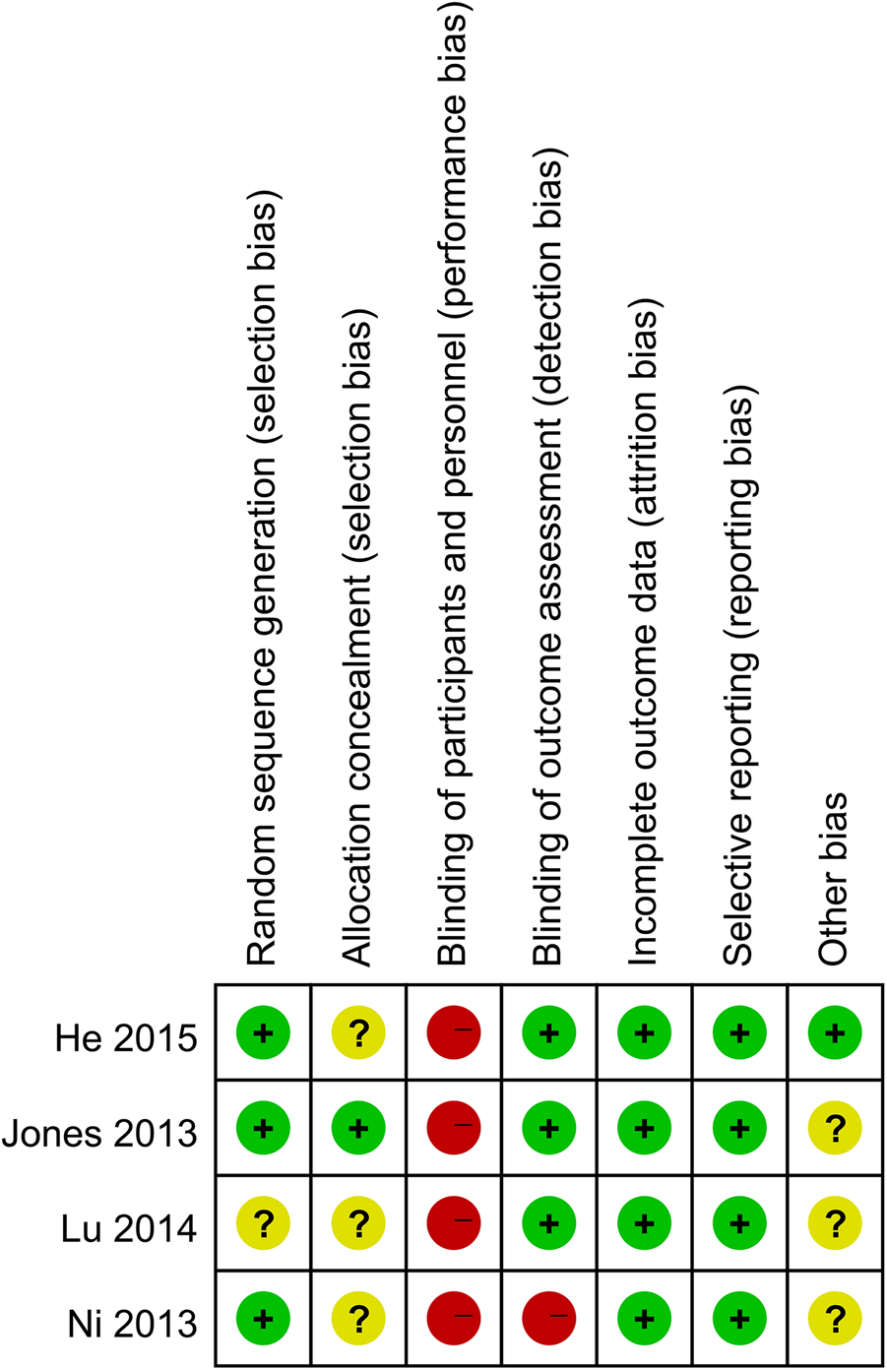
Risk of bias summary of all included studies

The RCT quality was determined using the RevMan bias assessment tool. This is shown in Figs. 2 and 3. Three studies reported randomization methods using random number generators (He et al. 2015; Jones et al. 2013; Ni et al. 2013). One study reported randomization without a detailed description (Lu et al. 2014). Only one study used sealed envelopes for allocation (Jones et al. 2013), but the other studies did not report allocation concealment. None of the RCTs blinded the patients or surgeons. Due to the nature of the ERAS programs, blinding of surgeons or patients is difficult. However, most of studies blinded the outcome assessors (He et al. 2015; Jones et al. 2013; Lu et al. 2014). All RCTs had a low risk of bias for incomplete outcome data and selective reporting. Other bias was unclear, except in one study (He et al. 2015).

### Meta-analysis

#### Primary outcome parameters

##### Mortality

All included studies reported on mortality (He et al. 2015; Jones et al. 2013; Lu et al. 2014; Ni et al. 2013), but the mortality in three studies was zero in both groups (He et al. 2015; Lu et al. 2014; Ni et al. 2013). No significant difference was found in the proportion of patients with mortality between the ERAS group (1/309; 0.3 %) and the conventional group (1/325; 0.3 %) (RR 0.98; 95 % CI 0.06–15.17; p = 0.99). As mortality was reported in only one study, the issue of fixed-effects model versus random-effects model does not exist.

##### Morbidity

**Fig. 4:**
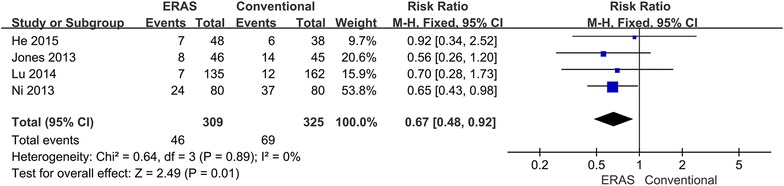
Comparison of overall complications between the ERAS group and the conventional group

All included studies reported on morbidity (He et al. 2015; Jones et al. 2013; Lu et al. 2014; Ni et al. 2013). In the ERAS group, 46 (14.9 %) patients developed overall morbidity, while 69 (21.2 %) patients in the conventional group sustained overall morbidity. The ERAS patients had a significant less overall morbidity (RR 0.67; 95 % CI 0.48–0.92; p = 0.01), with no heterogeneity between studies (p = 0.89, I^2^ = 0 %) (Fig. 4).

**Fig. 5:**
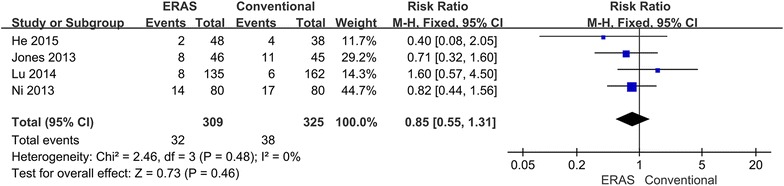
Comparison of major complications between the ERAS group and the conventional group

**Fig. 6:**
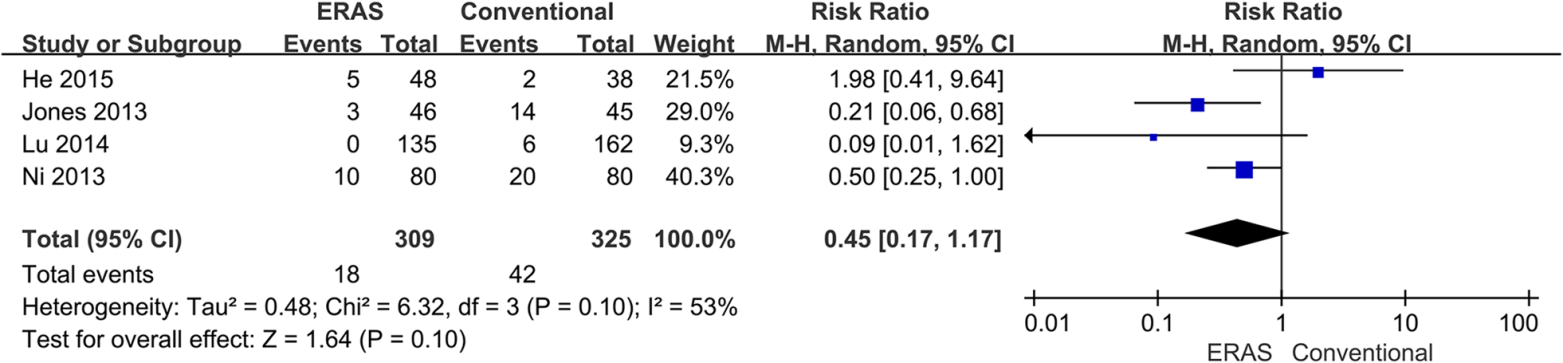
Comparison of minor complications between the ERAS group and the conventional group

Because different complications can cause varying degrees of risk, morbidity was divided into major and minor complications. Major complications appeared in 32 (10.4 %) ERAS patients and in 38 (11.7 %) conventional perioperative care patients (RR 0.85; 95 % CI 0.55–1.31; p = 0.46), with no heterogeneity between studies (p = 0.48, I^2^ = 0 %) (Fig. 5). Minor complications showed no difference between groups: 18 (5.8 %) ERAS patients versus 42 (12.9 %) conventional perioperative care patients (RR 0.45; 95 % CI 0.17–1.17; p = 0.10). However, some heterogeneity existed between studies (p = 0.10, I^2^ = 53 %). Therefore, the random-effects model was applied to this meta-analysis (Fig. 6). When the sensitivity analysis was restricted to the study (He et al. 2015), the heterogeneity decreased, and the ERAS patients had significantly reduced minor morbidity (RR 0.34; 95 % CI 0.15–0.73; p = 0.006).

##### Length of hospital stay

**Fig. 7:**
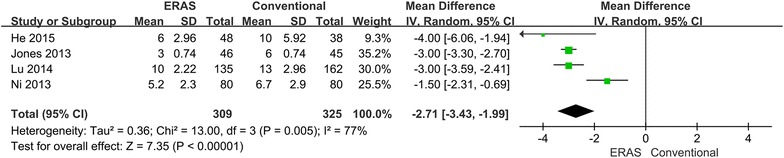
Comparison of primary length of stay between the ERAS group and the conventional group

**Fig. 8:**
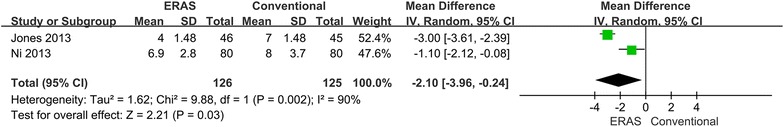
Comparison of total length of stay between the ERAS group and the conventional group

All included studies reported on primary length of stay (He et al. 2015; Jones et al. 2013; Lu et al. 2014; Ni et al. 2013), which was significantly shorter for the ERAS treated patients (WMD −2.71; 95 % CI −3.43 to −1.99; p < 0.00001). However, excessive heterogeneity existed between studies (p = 0.005, I^2^ = 77 %). Therefore, the random-effects model was applied (Fig. 7). When the sensitivity analysis was restricted to the study (Ni et al. 2013), the heterogeneity disappeared, but the results did not significantly change. Only two of included studies reported on the total length of stay (Jones et al. 2013; Ni et al. 2013), which was significant lower for the ERAS treated patients (WMD −2.10; 95 % CI −3.96 to −0.24; p = 0.03). Random-effect models were used to determine significant heterogeneity between studies (p = 0.002, I^2^ = 90 %) (Fig. 8).

##### Readmissions

**Fig. 9:**
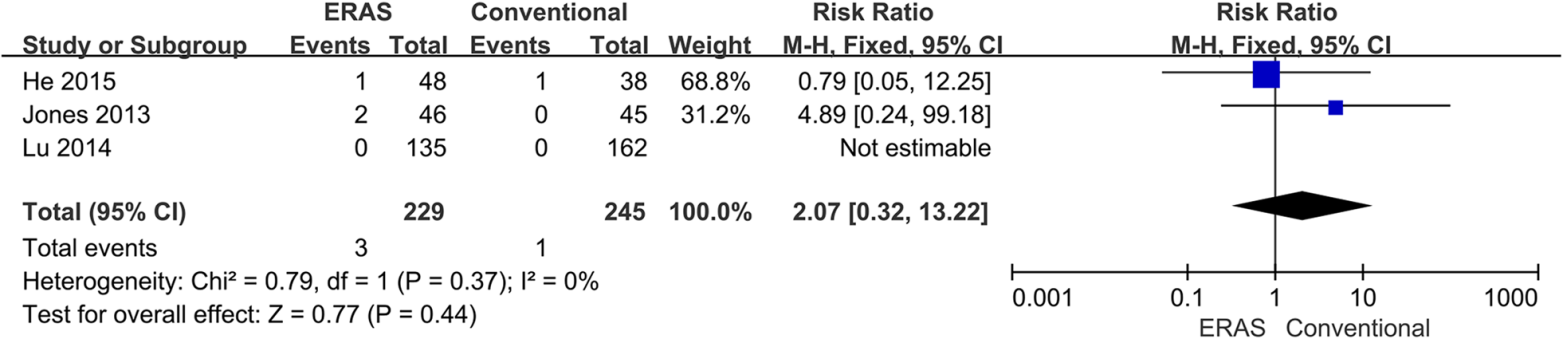
Comparison of readmissions between the ERAS group and the conventional group

Three of the included studies reported on readmissions (He et al. 2015; Jones et al. 2013; Lu et al. 2014). In total, three (1.3 %) patients in the ERAS group and one (0.4 %) patient from the conventional group were readmitted. No significant difference was found in readmissions between the two groups (RR 2.07; 95 % CI 0.32–13.22; p = 0.44), with no heterogeneity between studies (p = 0.37, I^2^ = 0 %) (Fig. 9).

##### Functional recovery

**Fig. 10:**
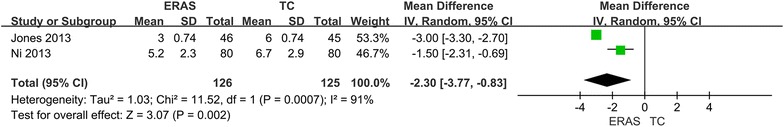
Comparison of time to functional recovery between the ERAS group and the conventional group

Only two of included studies reported on time to functional recovery (Ni et al. 2013; Jones et al. 2013), which was significantly shorter for the ERAS treated patients (WMD −2.30; 95 % CI −3.77 to −0.83; p = 0.002). The random-effects model was applied for excessive heterogeneity between studies (p = 0.0007, I^2^ = 91 %) (Fig. 10).

#### Secondary outcome parameters

##### Time to first flatus

**Fig. 11:**
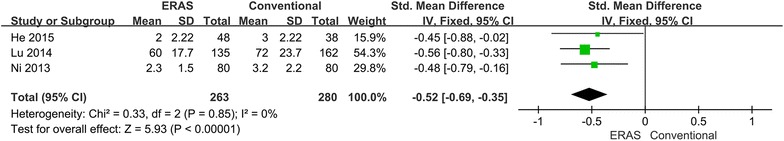
Comparison of time to first flatus between the ERAS group and the conventional group

Three studies reported on time to first flatus (He et al. 2015; Lu et al. 2014; Ni et al. 2013). After pooling the data, the ERAS patients had significantly shorter time to first flatus. (SMD, −0.52; 95 % CI −0.69 to −0.35; p < 0.00001), with no heterogeneity between studies (p = 0.85, I^2^ = 0 %) (Fig. 11).

##### Operative time

**Fig. 12:**
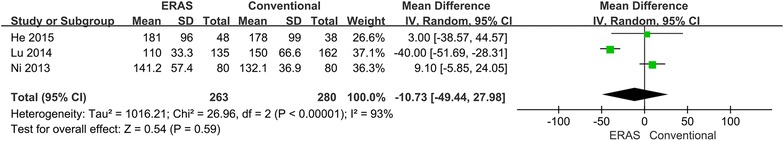
Comparison of operative time between the ERAS group and the conventional group

Three studies reported on operative time (He et al. 2015; Lu et al. 2014; Ni et al. 2013). No significant difference was noted between the ERAS group and the conventional group (WMD −10.73; 95 % CI −49.44 to 27.98; p = 0.59). The random-effects model was applied for excessive heterogeneity between studies (p < 0.00001, I^2^ = 93 %) (Fig. 12). When the sensitivity analysis was restricted to the study (Lu et al. 2014), the heterogeneity disappeared, but the results were the same.

##### Intraoperative blood loss

**Fig. 13:**
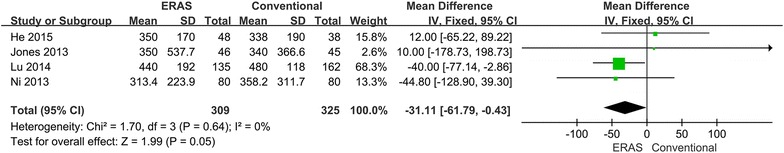
Comparison of intraoperative blood loss between the ERAS group and the conventional group

All included studies reported on intraoperative blood loss (He et al. 2015; Jones et al. 2013; Lu et al. 2014; Ni et al. 2013). No significant difference was noted for this outcome between the ERAS group and the conventional group (WMD −31.11; 95 % CI −61.79 to −0.43; p = 0.05), with no heterogeneity between studies (p = 0.64, I^2^ = 0 %) (Fig. 13).

##### Quality of life (QoL)

Because various questionnaires were used, we only performed a qualitative analysis on QoL.

Two of the included studies reported outcomes for QoL (He et al. 2015; Jones et al. 2013). He et al. used an ANOVA, but Jones et al. used the EQ-5D™. In the study by He et al., QoL was evaluated on 2, 4, 6, 8, 10, 20, 30, and 40 days after surgery. The ERAS patients received significantly higher scores, and the median area under a curve (AUC) was higher in the ERAS group. Jones et al. assessed QoL on POD 2, 3, 5, 7, 10, 14 and 28. The study showed that patients in the ERAS group had better QoL after 28 days (p = 0.002). The median AUC was also significantly higher in the ERAS group.

## Discussion

This meta-analysis compared the safety and efficacy of ERAS programs versus conventional care. The main outcome parameters reflect the safety of the intervention, which is always the greatest concern in clinical practice. Only one study reported on mortality, and no significant difference was noted between the two groups. The ERAS patients had a significant reduction in overall morbidity as well (Fig. 4). However, no significant difference in major (Fig. 5) and minor (Fig. 6) complications was found between groups. Therefore, the ERAS program is safe and does not compromise complications. However, the meta-analysis showed heterogeneity in minor morbidity. This may be because the recording methods and the definition of complications vary between studies. In addition, there is no clear distinction between major and minor complications. When a sensitivity analysis was performed, the heterogeneity disappeared.

A major problem was that the ERAS program might cause higher readmission rates. However, there was no difference between readmissions for the two procedures (Fig. 9). This might be because these studies used rigid and strict discharge criteria, which may be important indicators for speed of recovery. Both PHS and TLOS were significantly shorter in the ERAS group (Figs. 7, 8). This could be due to a high number of ERAS items. The analysis indicated that ERAS programs had an early discharge and did not increase the risk of readmission. However, it may not be an important outcome to provide quality care to patients because the factors that make patients able to or keen to leave hospital are many (Maessen et al. 2008). Additionally, there was higher heterogeneity in two outcome parameters, which may result from differences between studies in the implementation of ERAS programs and the number of ERAS items used. After performing a sensitivity analysis, the heterogeneity disappeared. The available evidence suggests that criteria of functional recovery are superior to length of stay for the evaluation of the success of an ERAS program (Maessen et al. 2008). The present study showed that time to functional recovery reduced in the ERAS group (Fig. 10). Therefore, the ERAS program can accelerate the postoperative recovery.

For secondary outcome parameters, ERAS programs also showed obvious advantages in time to first flatus and QoL. However, no significant difference was noted in operative time and intraoperative blood loss between groups.

### Quality of the evidence

The overall methodological quality was moderate, and most studies had at least one aspect of unclear or high risk of bias. Owing to the character of ERAS programs, no studies blinded the patients or surgeons. Not all outcome assessors were blinded, which may produce performance or measurement biases, especially in length of hospital stay. Only one study used allocation concealment (Jones et al. 2013), which may produce selection bias.

Another issue was the compliance with ERAS programs. Only two of the included studies reported the compliance with a fully implemented ERAS program (He et al. 2015; Jones et al. 2013). Therefore, the intervention effects may be enlarged or reduced. Additionally, there were no official statement or guidelines for ERAS programs in liver surgery in particular, and some useful ERAS items (especially epidural analgesia) were controversial, so only inspired guidelines of ERAS were applied by most centers. Although we had a limited the number of ERAS items, these effects still may be underestimated.

## Conclusions

This meta-analysis showed that ERAS programs appear to be safe and effective in liver surgery, significantly reduced overall morbidity rates, accelerated functional recovery, and shorten primary and total hospital stay, without compromising readmission rate. However, limitations include small sample sizes, methodological study quality, and the management of protocol compliance; therefore, the conclusions are limited. Further studies are needed to provide more solid evidence.

## References

[CR1] Bardram L, Funch-Jensen P, Jensen P, Crawford ME, Kehlet H (1995). Recovery after laparoscopic colonic surgery with epidural analgesia, and early oral nutrition and mobilisation. Lancet (London, England).

[CR2] Belghiti J, Kabbej M, Sauvanet A, Vilgrain V, Panis Y, Fekete F (1993). Drainage after elective hepatic resection. A randomized trial. Ann Surg.

[CR3] Coolsen MM, Wong-Lun-Hing EM, van Dam RM, van der Wilt AA, Slim K, Lassen K, Dejong CH (2013). A systematic review of outcomes in patients undergoing liver surgery in an enhanced recovery after surgery pathways. HPB.

[CR4] Dunne DFJ, Yip VS, Jones RP, McChesney EA, Lythgoe DT, Psarelli EE, Jones L, Lacasia-Purroy C, Malik HZ, Poston GJ, Fenwick SW (2014). Enhanced recovery in the resection of colorectal liver metastases. J Surg Oncol.

[CR5] Finch RJB, Malik HZ, Hamady ZZR, Al-Mukhtar A, Adair R, Prasad KR, Lodge JPA, Toogood GJ (2007). Effect of type of resection on outcome of hepatic resection for colorectal metastases. Br J Surg.

[CR6] He F, Lin X, Xie F, Huang Y, Yuan R (2015). The effect of enhanced recovery program for patients undergoing partial laparoscopic hepatectomy of liver cancer. Clin Transl Oncol.

[CR7] Higgins J, Green S (2011) Cochrane handbook for systematic reviews of interventions Version 5.1.0. [updated March 2011]. The Cochrane Collaboration, 2011. www.cochrane-handbook.org

[CR8] Higgins JP, Thompson SG (2002). Quantifying heterogeneity in a meta-analysis. Stat Med.

[CR9] Hozo SP, Djulbegovic B, Hozo I (2005). Estimating the mean and variance from the median, range, and the size of a sample. BMC Med Res Methodol.

[CR10] Hughes MJ, McNally S, Wigmore SJ (2014). Enhanced recovery following liver surgery: a systematic review and meta-analysis. HPB.

[CR11] Jones C, Kelliher L, Dickinson M, Riga A, Worthington T, Scott MJ, Vandrevala T, Fry CH, Karanjia N, Quiney N (2013). Randomized clinical trial on enhanced recovery versus standard care following open liver resection. Br J Surg.

[CR12] Jones EL, Wainwright TW, Foster JD, Smith JR, Middleton RG, Francis NK (2014). A systematic review of patient reported outcomes and patient experience in enhanced recovery after orthopaedic surgery. Ann R Coll Surg Engl.

[CR13] Kehlet H, Dahl JB (2003). Anaesthesia, surgery, and challenges in postoperative recovery. Lancet.

[CR14] Kehlet H, Wilmore DW (2002). Multimodal strategies to improve surgical outcome. Am J Surg.

[CR15] Koo V, Brace H, Shahzad A, Lynn N (2013). The challenges of implementing Enhanced Recovery Programme in urology. Int J Urol Nurs.

[CR16] Lassen K, Soop M, Nygren J, Cox PBW, Hendry PO, Spies C, von Meyenfeldt MF, Fearon KCH, Revhaug A, Norderval S, Ljungqvist O, Lobo DN, Dejong CHC (2009). Consensus review of optimal perioperative care in colorectal surgery. Arch Surg.

[CR17] Lei Q, Wang X, Tan S, Xia X, Zheng H, Wu C (2014). Fast-track programs versus traditional care in hepatectomy: a meta-analysis of randomized controlled trials. Dig Surg.

[CR18] Lu H, Fan Y, Zhang F, Li G, Zhang C, Lu L (2014). Fast-track surgery improves postoperative outcomes after hepatectomy. Hepatogastroenterology.

[CR19] Maessen JMC, Dejong CHC, Kessels AGH, von Meyenfeldt MF (2008). Length of stay: an inappropriate readout of the success of enhanced recovery programs. World J Surg.

[CR20] Nelson G, Kalogera E, Dowdy SC (2014). Enhanced recovery pathways in gynecologic oncology. Gynecol Oncol.

[CR21] Ni CY, Yang Y, Chang YQ, Cai H, Xu B, Yang F, Lau WY, Wang ZH, Zhou WP (2013). Fast-track surgery improves postoperative recovery in patients undergoing partial hepatectomy for primary liver cancer: a prospective randomized controlled trial. Eur J Surg Oncol.

[CR22] Reissfelder C, Rahbari NN, Koch M, Kofler B, Sutedja N, Elbers H, Buchler MW, Weitz J (2011). Postoperative course and clinical significance of biochemical blood tests following hepatic resection. Br J Surg.

[CR23] Sánchez-Pérez B, Aranda-Narváez JM, Suárez-Muñoz MA, elAdel-delFresno M, Fernández-Aguilar JL, Pérez-Daga JA, Pulido-Roa Y, Santoyo-Santoyo J (2012). Fast-track program in laparoscopic liver surgery: theory or fact?. World J Gastrointest Surg.

[CR24] Spanjersberg WR, Reurings J, Keus F, van Laarhoven CJ (2011). Fast track surgery versus conventional recovery strategies for colorectal surgery. Cochrane Database Syst Rev.

[CR25] van Dam RM, Wong-Lun-Hing EM, van Breukelen GJ, Stoot JH, van der Vorst JR, Bemelmans MH, Olde Damink SW, Lassen K, Dejong CH (2012). Open versus laparoscopic left lateral hepatic sectionectomy within an enhanced recovery ERAS^®^ programme (ORANGE II—Trial): study protocol for a randomised controlled trial. Trials (Electronic Resource).

